# Hybrid and Deep Learning Approach for Early Diagnosis of Lower Gastrointestinal Diseases

**DOI:** 10.3390/s22114079

**Published:** 2022-05-27

**Authors:** Suliman Mohamed Fati, Ebrahim Mohammed Senan, Ahmad Taher Azar

**Affiliations:** 1 College of Computer and Information Sciences, Prince Sultan University, Riyadh 11586, Saudi Arabia; aazar@psu.edu.sa; 2 Department of Computer Science & Information Technology, Dr. Babasaheb Ambedkar Marathwada University, Aurangabad 431004, India; senan1710@gmail.com; 3 Faculty of Computers and Artificial Intelligence, Benha University, Benha 13518, Egypt

**Keywords:** deep learning, hybrid techniques, neural network, gastrointestinal diseases, LBP, GLCM, FCH, endoscope

## Abstract

Every year, nearly two million people die as a result of gastrointestinal (GI) disorders. Lower gastrointestinal tract tumors are one of the leading causes of death worldwide. Thus, early detection of the type of tumor is of great importance in the survival of patients. Additionally, removing benign tumors in their early stages has more risks than benefits. Video endoscopy technology is essential for imaging the GI tract and identifying disorders such as bleeding, ulcers, polyps, and malignant tumors. Videography generates 5000 frames, which require extensive analysis and take a long time to follow all frames. Thus, artificial intelligence techniques, which have a higher ability to diagnose and assist physicians in making accurate diagnostic decisions, solve these challenges. In this study, many multi-methodologies were developed, where the work was divided into four proposed systems; each system has more than one diagnostic method. The first proposed system utilizes artificial neural networks (ANN) and feed-forward neural networks (FFNN) algorithms based on extracting hybrid features by three algorithms: local binary pattern (LBP), gray level co-occurrence matrix (GLCM), and fuzzy color histogram (FCH) algorithms. The second proposed system uses pre-trained CNN models which are the GoogLeNet and AlexNet based on the extraction of deep feature maps and their classification with high accuracy. The third proposed method uses hybrid techniques consisting of two blocks: the first block of CNN models (GoogLeNet and AlexNet) to extract feature maps; the second block is the support vector machine (SVM) algorithm for classifying deep feature maps. The fourth proposed system uses ANN and FFNN based on the hybrid features between CNN models (GoogLeNet and AlexNet) and LBP, GLCM and FCH algorithms. All the proposed systems achieved superior results in diagnosing endoscopic images for the early detection of lower gastrointestinal diseases. All systems produced promising results; the FFNN classifier based on the hybrid features extracted by GoogLeNet, LBP, GLCM and FCH achieved an accuracy of 99.3%, precision of 99.2%, sensitivity of 99%, specificity of 100%, and AUC of 99.87%.

## 1. Introduction

Cancer is the greatest threat to human life and is the world’s second leading cause of death, following heart disease and atherosclerosis. There are numerous upper and lower gastrointestinal malignancies. Upper gastrointestinal cancers include malignancies of the esophagus and stomach, whereas lower gastrointestinal cancers include colon and rectal cancers. According to a World Health Organization estimate, 3.5 million instances of GI cancer were registered in 2018. The least common type of gastrointestinal cancer is esophageal cancer, but stomach cancer is the fifth most prevalent type of cancer and the third leading cause of death. In contrast, lower-GI tumors are the third most common cancer and the second most common cause of death [1]. Gastrointestinal diseases vary between ulcers, bleeding and polyp, which require early diagnosis otherwise they will develop and be a cause of death. There are many biomarkers to predict health problems in the gastrointestinal tract. However, the high mortality rate shows that there are still possibilities for early diagnosis to receive treatment on time and reduce side effects. If polyps are not diagnosed early, they turn into gastrointestinal cancer [2], which is abnormal cells on the mucous membrane of the colon and stomach. Polyps grow very slowly, and symptoms do not appear until significant [3]. Endoscopy devices that cause pain were used to detect polyps, bleeding and ulcers, and the results of their analysis were inaccurate due to their complex structure [4]. This challenge was overcome in 2000 by using new endoscopic techniques called wireless capsule endoscopy (WCE). WCE is a modern method for detecting and diagnosing diseases of the GI, which has the ability to scan the GI as a whole from the esophagus to the colon in a large video that is divided into frames of thousands of images. Therefore, this massive number of pictures poses a challenge for manual diagnosis because polyps, bleeding, and ulcers may appear in very few frames. In contrast, a massive number of frames look normal. Therefore, all frames must be carefully monitored by experts and doctors, which may take two hours or more to make a proper diagnosis. The WCE technology detects many serious diseases such as polyps, bleeding and ulcers. The large WCE images represent a burden on the gastroenterologist, as it is challenging to track all the images. The morphological features of each disease vary in terms of shape, color, and structure, as well as the etiology of each disease. Each disease has anatomical features that can be distinguished through the endoscope. The endoscope detects the type and location of the disease and gives a brief description and detailed documentation of the most important anatomical landmarks. Additionally, the pathological finding considers abnormal features in the gastrointestinal tract, which can be detected by endoscopy, as a change in the mucous membrane. These signs may be polyps or malignant tumors or persistent disease. Therefore, the early diagnosis of diseases such as polyps and tumors by endoscopy is important in receiving appropriate treatment and survival. Additionally, manual diagnosis is a tedious task that requires tracking all video frames and high experience and clinical knowledge. These challenges can be solved by developing effective computer-aided diagnostic systems represented by machine, deep learning and hybrid techniques. These techniques can assist physicians in deciding on an appropriate diagnosis during the initial stage of the disease [5].

Artificial intelligence systems have shown enormous promise in diagnosing medical images, assisting doctors and specialists in visualizing minute details that the naked eye cannot see [6]. Endoscopic images are used to extract subtle and complicated information using these techniques. They can also distinguish between malignant (neoplastic) and benign tissues. Machine learning approaches can also extract texture, color, form, and edge data with high accuracy and classify all endoscopic pictures according to the type of disease they represent. [7]. Convolutional Neural Networks have proven their great ability to extract feature maps and solve all geometrical feature constraints, which led to accurate diagnosis of GI images. The hybrid technique between machine and deep learning has superior advantages in extracting the deep features by CNN models and sorting them with great speed and accuracy by machine learning algorithms. Thus, artificial intelligence techniques have proven their superiority over the best-specialized experts [8]. ReedT et al. proposed artificial intelligence methods for diagnosing endoscopic images taken from the HyperKvasir dataset. They used grid search to set the best parameters by checking the intersection [9]. Sebastian et al. focused on the detection of polyps in the colon using artificial intelligence techniques that help doctors distinguish the type of tumor [10]. Sharib Ali et al. Due to the presence of artifacts in the internal endoscopy images, which hinder deep learning models for accurate diagnosis. Thus, the researchers focused on removing artifacts, segmentation the lesion area (EAD2020), and detecting the type of disease (EDD2020) [11].

The main contributions of this study are as follows:

- Enhance images using overlapping filters to remove noise, increase contrast, and reveal the edges of the lesion.
- Extracting features by using a hybrid method between three algorithms: LBP, GLCM and FCH.
- Applied hybrid technology consisting of two blocks: the first block is CNN models for extracting feature maps, and the second block is the SVM algorithm for classifying feature maps.
- Fusing features extracted by both CNN models and traditional algorithms (LBP, GLCM, and FCH) to form feature vectors that are more representative of each disease.
- The development of many proposed systems to assist physicians and endoscopy specialists in supporting their diagnostic decisions.

The remainder of the paper is organized as follows: Section 2 reviews the relevant previous studies. Section 3 includes several of methodologies for analyzing and diagnosing the lower gastrointestinal disease dataset. Section 4 presents the experimental results for evaluating the dataset on the proposed systems. Section 5 summarizes the discussion and comparison of the proposed approaches’ performance. Section 6 concludes the paper with future directions.

## 2. Related Work

This section will go over a number of prior studies on the diagnosis of gastrointestinal diseases. In addition, we will develop our technique and vary our methods and materials in order to get superior results in identifying lower GI disorders.

Akshay et al. proposed a colon cancer diagnosis system. The noise was removed to improve the images. Each image was divided into several blocks, and each block was diagnosed using CNN and machine learning algorithms. The system achieved 87% accuracy with the CNN model and 83% accuracy with the KNN algorithm [12]. Tsuyoshi et al. presented a CNN model called Single Shot MultiBox Detector to evaluate the colon dataset. The model was trained on 16,418 images and tested on 7077 images. Each frame was processed for twenty seconds, and the model achieved a sensitivity of 92%, and adenomas were detected with an accuracy of 97% [13]. Alexandr et al. suggested a methodology for early detection of polyps; The system goes through two stages: First, based on the universal features, polyps are classified as having a tumor or not. In the second stage, using CNN models for lesion segmentation, the system achieved a sensitivity and specificity of 93% and 82%, respectively [14]. Ruikai et al. developed a methodology to detect polyps in the colon and rectum. The system worked to identify polyps and predict polyps. The method achieved an accuracy of 87.3% compared to an accuracy of 86.4% by endoscopic specialists [15]. Eduardo et al. presented the CNN model of colonic mucosa diagnosis for colonic polyps. The network extracts and classifies features by exploiting the input pixels and optimizing all noise [16]. Min et al. developed an Asymptotic Laplacian-energy-like invariant of lattices of a system that analyzes the color of the lesions to predict polyps. The dataset was divided into 108 images for training the system and 181 images for testing the system and compared with the results of endoscopy specialists. The system reached an accuracy of 87% during the training phase, and the system achieved an accuracy of 78.4% during the testing phase, compared to an accuracy of 79.6% by endoscopy specialists [17]. Eun et al. Developed an automated computer-aided system to predict the types of diseases in the colon and rectum using CNN models. The system distinguishes between three polyps: serrated polyp (SP), benign adenoma (BA), and deep submucosal cancer (DSMC). The system achieved the highest accuracy of 82.4% [18]. Mehshan et al. presented a method based on deep learning for diagnosing gastrointestinal diseases. The lesion area was segmented by the modified mask and isolated from the rest of the image. ResNet101’s pre-trained model is configured to extract the most critical features and categorize them by MSVM [19]. Mustaine et al. discussed improving the way polyps are detected and helping clinicians focus on the most critical areas to diagnose. Colored waveforms and features by a CNN model were extracted to train an SVM classifier. SVM works to see if the images contain a tumor or not [20].

Mahmodul et al. presented a method for polyp diagnosis by fusion of CNN model and contour transformation. Dimensions were reduced and the most important features were combined by Minimum Redundancy Maximum Relevance (MRMR) and Principal Component Analysis (PCA) methods [21]. Chathurika et al. presented a method for integrating the deep features extracted from three CNN models and pooling them into a global average pooling (GAP) layer. The method achieved promising results in the diagnosis of gastrointestinal diseases [22]. Jasper et al. proposed a methodology for evaluating endoscopic images of lesions, including the size and location of the lesion. The method also helps assess the surface pattern and the possibility of excision of the lesion by endoscopic [23]. Roger et al. extracted features by the Global Average Pooling (GAP) to distinguish tumors. Data augmentation technique was applied to balance the dataset; the method reached good results [24]. Şaban et al. provided CNN models to classify the GI dataset; pooling layer features were transferred to the LSTM layer, then all LSTM layers were collected to classify each image [25]. Maghsoudi et al. presented a model for segmentation of polyps of endoscopic images using simple linear iterative clustering (SLIC). The highest sensitivity is found by examining SLIC super-pixels and then classifying them by the SVM classifier, which reached a sensitivity of 91% [26].

Jeph Herrin et al. presented three machine learning algorithms, namely, random survival forests, XGBoost, and RegCox, to predict gastrointestinal bleeding to help clinicians make their decisions. The performance of machine learning algorithms for predicting gastrointestinal bleeding was evaluated using accuracy, sensitivity, and AUC measures. The RegCox algorithm has an AUC of 67%, better than the others [27]. Jayeshkumar et al. presented a random forest algorithm for diagnosing gastrointestinal disorders. The study was conducted on a group of older people with osteoporosis and extract the features. The features were fed to the random forest to diagnose people with gastrointestinal disorders [28]. Hye Jinet al. suggested CAD models for diagnosing gastrointestinal lesions by endoscopic images. Image optimization, noise removal, lesion area segmentation, essential feature extraction, then classification was carried out by machine learning algorithms. The system achieved a sensitivity of 86%, a specificity of 90%, and an AUC of 94% [29].

Previous studies contain drawbacks when applying deep learning models, which are very time-consuming when training the dataset and require costly computers and the inability of the systems to reach the required accuracy. For machine learning algorithms, the drawbacks are that they cannot train a huge dataset. Thus, these obstacles were overcome in this work using hybrid techniques between deep learning to extract feature maps and classify them by the SVM algorithm. Additionally, features from three algorithms were extracted and combined into one feature vector, in addition to the application of a novel method, which is a hybrid technology for extracting features in a hybrid way between deep learning models and GLCM, LBP, and FCH algorithms, then integrating all the features in one vector and classifying them using neural networks, which reached promising results.

## 3. Materials and Methodology

**Figure 1:**
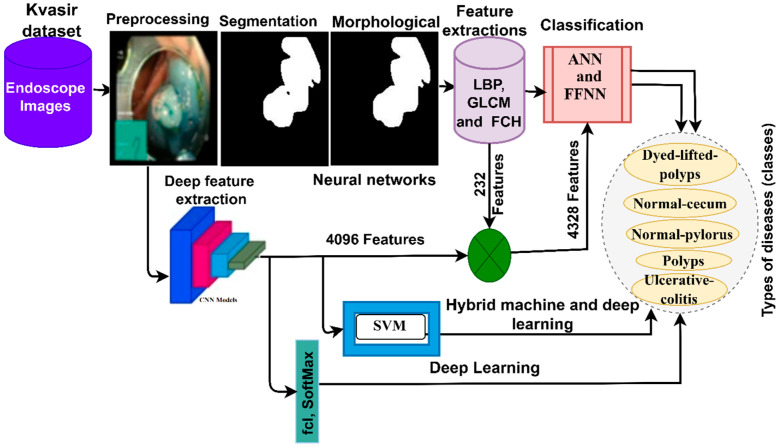
The general methodology for diagnosing lower GI disease dataset.

In this section, the methodology and materials are presented to diagnose endoscopic images for the early detection of lower gastrointestinal diseases, as shown in Figure 1. The first step was to optimize all the images to remove noise and increase the contrast of the edges. Then, the optimized images were fed into four suggested methods. The first method is to classify a dataset using ANN and FFNN based on the hybrid features between LBP, GLCM and FCH algorithms. The second method is to diagnose a dataset by the CNN models GoogLeNet and AlexNet. The third method uses a hybrid technique between CNN models and machine learning (SVM) to diagnose the dataset. The fourth method of dataset diagnosis uses ANN and FFNN based on hybrid features extracted from CNN models and LBP, GLCM and FCH algorithms.

### 3.1. Description of the Dataset

**Figure 2:**
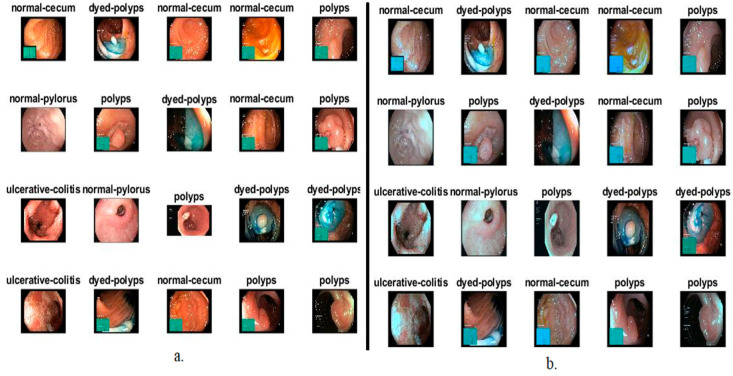
Images of the dataset for all types of diseases (**a**) Before enhancement (**b**) After enhancement.

The Kvasir dataset was obtained using high-resolution endoscopic cameras by Vestre Viken Health Trust (VV) from the Department of Lower GI, Bærum Hospital, Gjettum, Norway. All images were described by several experts from the Cancer Registry of Norway (CRN) and VV. The CRN is the national body at the University of Oslo Teaching Hospital, responsible for the diagnosis and early detection of cancer to prevent its spread. CRN provides knowledge about cancer and is affiliated with the southeast Norway Health Authority under the supervision of Oslo University Hospital. CRN is responsible for detecting precancerous lesions to prevent death by cancer. All images were described by medical experts, including anatomical landmarks, pathological findings, and regular findings. The dataset contains 5000 images evenly distributed across five classes of diseases: dyed-lifted polyps, normal cecum, normal pylori, polyps, and ulcerative colitis. All images are in RGB color space and have resolutions ranging from 700 × 575 to 1925 × 1075 pixels. Anatomical landmarks include the cecum and pylorus, while pathological findings include polyps and ulcerative colitis, in addition to other images related to removing lesions, such as a lifted polyp. Some image classes contain green images showing the location of the endoscope of the bowel through the electromagnetic imaging system. Figure 2a describes a dataset sample for all the classes contained in the Kvasir dataset [30]. The following link is open source and includes the dataset https://datasets.simula.no/kvasir/#download (accessed on 30 January 2022).

### 3.2. Images Enhancement

When using endoscopes to perform interior imaging, several restrictions and obstructions appear as artifacts owing to movement, bubbles, low contrast, bodily fluids, debris, residual feces, and blood. These artifacts impede image classification; hence, all artifacts must be removed to achieve superior outcomes in the subsequent stages of medical image processing. All photos in our study were optimized before being given to the subsequent stages: the dataset images were optimized by averaging RGB colors and passing images through both average and Laplacian filters for removing artifacts, improving image contrast, and showing lesion edges [31]. First, a 5 × 5-pixel averaging filter is defined which removes artefacts and increases image contrast by replacing each central pixel with an average of 24 adjacent pixels. The filter in each iteration works by taking a center pixel and replacing the central pixel’s value with the average value of 24 adjacent pixels. Equation (1) describes how the average filter works, and the process continues until the last pixel of the image [32].

(1) $$\;F\left(L\right)=\frac{1}{L}\overset{L-1}{\underset{i=0}{\sum }}z\left(L-1\right)\;$$

where *F (L)* refer to the enhanced image, *z*(*L* − 1) refers to the input of previous, and *L* refers the pixel’s number in the image.

Secondly, the Laplacian filter is applied which makes the edges of the tumors visible and distinguishes them from the rest of the image. Equation (2) describes the filter’s mechanism of action.

(2) $$\nabla {\;}^{2}\;f=\frac{d{\;}^{2}\;f}{d^{\;2}\;x}+\frac{d^{2}\;f}{d^{\;2}\;y}\;$$

where *x*, *y* are to the 2D matrices and $\nabla {\;}^{2}\;f$ is a second-order differential equation.

Finally, the two images are merged together by subtracting the image generated by the average filter from the image generated by the Laplacian filter as shown in Equation (3).

(3) $$O\left(X\right)=f\left(L\right)-\nabla {\;}^{2}\;f\;$$

where O(X) represents output image enhanced.

Thus, an improved image is obtained that is fed to all the proposed systems in this study. Figure 2b describes many image samples for all diseases of the dataset after enhancement operations.

### 3.3. The First Proposed System (Neural Networks)

#### 3.3.1. Segmentation (Active Contour Algorithm)

All lower digestive system endoscopy images contain a specific region affected by a lesion and the rest of the image is healthy. Therefore, analyzing the entire image, including the healthy part, and extracting the features from the complete image will lead to incorrect diagnostic results. Thus, the segmentation technique is necessary to segment the lesion area (area of interest) and isolate it from the healthy part, which will lead to the analysis and extraction of features from the lesion area only to obtain promising diagnostic results.

The lesion area is determined by the curve ∁ defined by the level function ∅: Ω *→,* where Ω represents the lesion area, and zero is at the first border area in the image of the lower gastrointestinal lesion 𝐼. The curve is divided of lesion regions $F_{k}$ ⊂Ω into sub-regions *F*, *F* with ϕ, as shown in Equations (4) and (5).

(4) $$\mathrm{inside}\;\left(∁\right)=F=\left\{\;x\;\in \;F_{k}:\;\emptyset \left(x\right)>0\right\}\;$$

(5) $$\mathrm{outside}\;\left(∁\right)=F=\left\{\;x\;\in \;\Omega \;:\;\emptyset \left(x\right)<0\;\cup x\;\in \;\Omega \;\backslash F_{k}\right\}\;$$

The Active Contour algorithm develops by moving the curve (contour) inward. The first seed is placed at the lesion boundary to map the lesion area (polyp). The curve moves inside to define the polyp subregion when ϕ > 0 is set. The outer region is determined by subtracting the formerly selected region from the presently selected region as described in Equation (6).

(6) $$\begin{array}{c} F_{0}\;=\;F_{1}+{\bar{F}}_{1}\;\Rightarrow \;{\bar{F}}_{1}\;=\;F_{0}\;-\;F_{1},\\ F_{2}\;=\;F_{1}-F_{2},\\ {\bar{F}}_{3}\;=\;F_{2}\;-\;F_{3}. \end{array}$$

Finally, the outer sub-region can be calculated as Equation (7).

(7) $${\bar{F}}_{k}\;=\;F_{k\;-\;1}\;-\;F_{k}$$

The Active Contour moves toward the lesion boundary by defining the external energy that moves the first point (the zero level) toward the lesion boundary, as described by the functional energy function Equation (8).

(8) $$f_{spz}\left(\emptyset \right)=\lambda L_{spz}\left(\emptyset \right)+\nu \;A_{spz}\left(\emptyset \right)\;$$

where ν are constants and λ > 0. The $L_{spz}\;and\;A_{spz}$ are the defined as Equations (9) and (10).

(9) $$L_{spz}\left(\emptyset \right)=\int_{\Omega }spz\left(I\right)\delta_{\varepsilon }\left(\emptyset \right)\;\left|\nabla \emptyset \right|\;dx$$

(10) $$A_{spz}\left(\emptyset \right)=\int_{\Omega }spz\left(I\right)H_{\varepsilon }\left(\emptyset \right)\;\left(-\emptyset \right)\;dx\;$$

the term $spz\left(I\right)$ will be defined in an equation later, 𝐻𝜀 denotes the Heaviside function and $\delta_{\varepsilon }$ is the univariate dirac delta function. The curve is driven from zero point to smooth curve by *Lspz*(ϕ) equation. Thus, the small energy *spz(I)* will speeds up the curve towards the lesion. The coefficient *v* is a positive or negative value that depends on the position of the curve over the lesion area; If the curve is within the lesion region, *v* is positive while *v* is negative for acceleration of the curve at the lesion boundary [33].

We proposed the E_proposed_ for the *SPF* function which previously used. Let 𝐼: Ω → 𝑅 be endoscopic image of lower gastrointestinal diseases, *C* is a closed curve energy functional is defined as by Equation (11).

(11) $$E_{\mathrm{proposed}}=\int_{\Omega }\;{\left|I\left(x\right)-C_{1}\right|}^{2}H_{\varepsilon }\left(\emptyset \left(x\right)\right)M^{k}\left(x\right)\;\;dx+\int_{\Omega }\;{\left|I\left(x\right)-C_{1}\right|}^{2}\;(1-H_{\varepsilon }\left(\emptyset \left(x\right)\right))M^{k}\left(x\right)\;\;dx\;\;$$

when the energy $E_{\mathrm{proposed}}$ is reduced, ϕ is preserved constant, we get the curve *C*_1_ for *F* region w and the curve *C*_1_ for area $\bar{F}$ as Equations (12) and (13).

(12) $$C_{1}\left(\phi \right)=\frac{\int_{\Omega }\;I\left(x\right)\;H_{\varepsilon }\left(\emptyset \left(x\right)\right)M^{k}\left(x\right)\;\;dx}{\int_{\Omega }\;H_{\varepsilon }\left(\emptyset \left(x\right)\right)M^{k}\left(x\right)\;\;dx}$$

(13) $$C_{2}\left(\phi \right)=\frac{\int_{\Omega }\;I\left(x\right)\;(1-H_{\varepsilon }\left(\emptyset \left(x\right)\right))M^{k}\left(x\right)\;\;dx}{\int_{\Omega }\;(1-H_{\varepsilon }\left(\emptyset \left(x\right)\right))M^{k}\left(x\right)\;dx}$$

$M^{k}$ refers to the function of characteristic for sub-region as Equation (14).

(14) $$M^{k}\left(x\right)=\phi >0,$$

$$M^{0}:\;\Omega \;\to -1,$$

The contour curve continues to move along the edges of the lesion, and when the pixels are similar between two successive contours, the curve will stop, and the algorithm stops by a certain stop value.

If

(15) $$\sum_{i=0}^{row}\overset{col}{\underset{j=0}{\sum }}M_{i,j}^{k}<\left(\frac{\mathrm{SV}}{100}\right)\;\sum_{i=0}^{row}\overset{col}{\underset{j=0}{\sum }}oldM_{i,j}^{k}$$

where SV represents the stop value when the curve reaches the last point to separate the pest region from the rest of the image.

Then, the contour curve will be stopping moving.

Where$\;oldM_{i,j}^{k}$ refers to the last computed mask, and $M_{i,j}^{k}\;$refers to the current mask of the contour curve, col and row are the max number of columns and rows, respectively, in the lesion.

**Figure 3:**
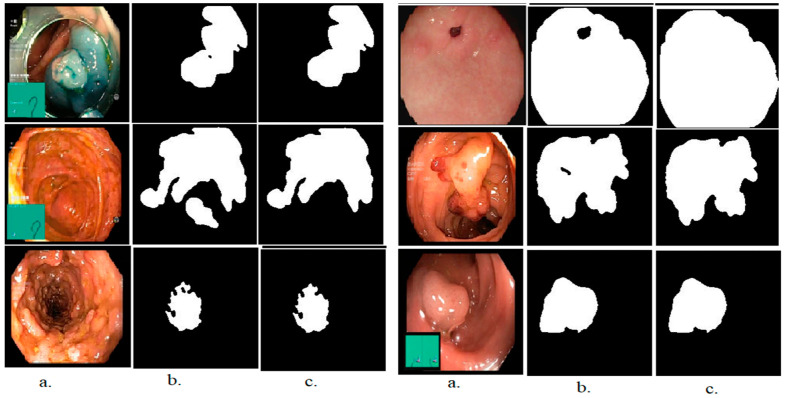
Samples of the dataset (**a**) original image (**b**) After the segmentation (**c**) After the morphological.

Finally, the polyp lesion area is identified with high accuracy and isolated from the rest of the image, as shown in Figure 3b. After the segmentation process, a binary image of the lesion area is produced and isolated from the rest of the image. Still, some holes appear that do not belong to the lesion, and therefore these holes must be filled to improve the image [34]. Thus, the morphological method was applied, which produces an enhanced binary image, as shown in Figure 3c.

#### 3.3.2. Feature Extraction

The feature extraction stage is one of the most critical stages in image processing, which determines the accuracy and efficiency of classification. The image contains thousands of information that is difficult to analyze. Therefore, extracting representative features with high accuracy reduces image dimensions and extracts features from a region of interest (ROI). In this work, the features were extracted by three algorithms: LBP, GLCM and FCH. The features extracted from the three methods are then combined into a single feature vector for each image to obtain more representative features. Combining features is a modern and important method in obtaining more effective and representative features for each lesion.

First, the LBP algorithm extracts the essential features, which is an efficient way to extract the texture features of the binary surface. In this work, an algorithm was tuned on size of 6 × 6; it works to select the central pixel (*g_c_*) and select the 35 pixels adjacent to it (*g_p_*) [35]. According to the algorithm, the central pixel is replaced by neighboring pixels in each iteration. Neighboring pixels are selected according to the radius R. Equations (16) and (17) describe the working mechanism of the LBP algorithm, which replaces the central pixel with adjacent pixels and continues until all pixels of the image are targeted. This algorithm extracted 203 essential features for each image, stored in one attribute vector for each image.

(16) $$LBP\;{\left(x_{c},\;y_{c}\right)}_{R,P}=\overset{P-1}{\underset{P=0}{\sum }}s\left(g_{p}-g_{c}\right)\;2^{P}$$

(17) $$x\left(c\right)=\left\{\begin{array}{c} 0,\;c<0\\ 1,\;c\geq 0 \end{array}\right.\;$$

*P* is the number of pixels in the image and *R* is the number of adjacent pixels.

Second, the GLCM algorithm extracted the features, which extracts texture features from the area of interest (tumors).

The algorithm shows multiple levels in the grayscale of an area of interest. The algorithm extracts the statistical features based on its working mechanism. The algorithm relies on distinguishing between smooth and rough pixels through spatial information. An area is rough when its pixel values are far apart, while when the pixel values are close together, the area is smooth. Additionally, spatial information is essential in determining the correlation between pairs of pixels through the distance d between the pixels and the directions θ that determine the location of each pixel from the other [36]. There are four directions for θ, which are 0°, 45°, 90° and 135°; when the angle is vertical θ = 90 or horizontal θ = 0, the distance between one pixel and the other d = 1. When the angle θ between pair pixel θ = 45 or θ = 135, the distance d = √2. This algorithm generated 13 statistical features for each image.

Third, the features are extracted from a region of interest by the FCH algorithm. FCH is considered one of the best algorithms for extracting the chromatic features from the pest area. The color is one of the most important features used in medical images to distinguish and diagnose images of gastrointestinal tumors [37]. The lesion region contains many colors, so each color is represented in the histogram bin. Each color in the region of interest represents several histogram bins. Each local color is represented in histogram bin and thus pest colors are distributed over many histogram bins. All two similar colors must be in the same histogram bin, also if the two colors in the histogram bin are different, it means that they are different even if they are almost the same. Thus, the FCH algorithm uses the membership value of each pixel and distributes the pixels based on the total histogram bin. We consider the number of colors in a lesion area containing *n* pixels as $X\left(I\right)=x_{1,}x_{2,}\ldots x_{i}$ where *n_i_* is the image pixels, ith is the all-color bins, $x_{i=ni/n}\;$is the probability that any color image belongs to a histogram bin.

(18) $$x_{i}=\overset{n}{\underset{j=1}{\sum }}p_{i/j}\;p_{j=}\frac{1}{n}\;\overset{n}{\underset{j=1}{\sum }}p_{i/j}\;$$

where *p_j_* is image pixels, conditional probability $p_{i/j}$, means the probability of jth pixel belonging to *I* histogram color bins using the FCH algorithm.

(19) $$p_{i/j}=\left\{\begin{array}{c} 1,\;\;\;\;\;\;\;\;\;\mathrm{if}\;\;\;jth\;\;\mathrm{pixels}\;\mathrm{belongs}\;\mathrm{to}\;ith\;\mathrm{histogram}\;\mathrm{color}\;\mathrm{bin}\\ 0\;,\;\;\;\;\;\;\;\;\;\;\;\;\;\;\;\;\;\;\;\;\;\;\;\;\;\;\;\;\;\;\;\;\;\;\;\;\;\;\;\;\;\;\;\;\;\;\;\;\;\;\;\;\;\;\;\;\;\;\;\;\;\;\;\;\;\;\;\;\;\;\;\;\;\;\;\;\;\;\;\mathrm{Otherwise} \end{array}\right.$$

**Figure 4:**
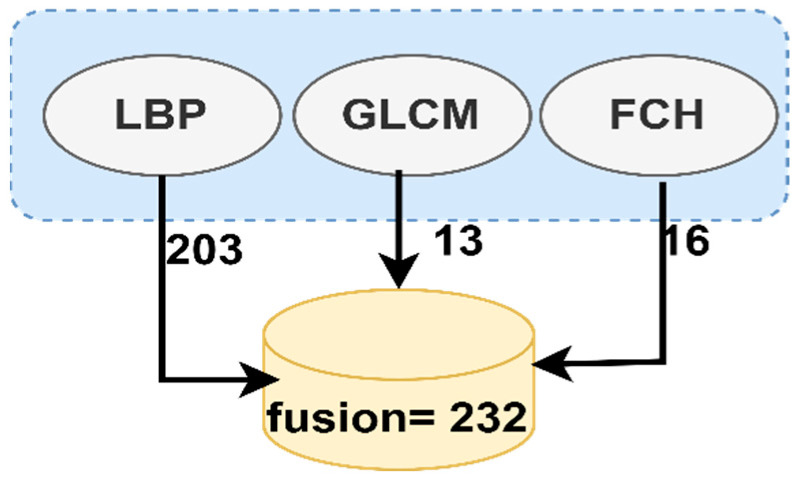
The fusion method between the three algorithms LBP, GLCM and FCH.

Finally, the features extracted from the three algorithms are combined into one feature vector for each image. The LBP method produce of 203 features, 13 features produced from the GLCM method, and the FCH method produce 16 features. Therefore, when all the features were hybrid, the Fusion method produced 232 representative features for each image, representing each gastrointestinal polyp’s lesion’s essential and distinguishing features. Figure 4 describes the fusion method between the three algorithms, LBP, GLCM and FCH.

#### 3.3.3. ANN and FFNN Algorithms

In this section, the endoscopic dataset for lower GI is evaluated using the ANN and FFNN neural network algorithms. ANN is a type of intelligent neural network that consists of three main layers, each layer having many interconnected neurons. ANN is characterized by its profound ability to solve many complex problems by analyzing, interpreting and transforming complex data into information to solve the required tasks. ANN consists of three layers: the input layer, the hidden layers, and the output layer. Data moves from one layer to another through connections called weights. The first layer of the algorithm is the input layer, which receives information from (the features extracted) and passes it to the hidden layer. In our study, the number of neurons in the input layer is 232 neurons (features extracted). Hidden layers solve complex problems and analyze and interpret inputs across many hidden layers, and each hidden layer has many interconnected neurons. In our study, the hidden layers were set to 30 hidden layers. The output layer receives the output from the last hidden layer and consists of many neurons according to the classes of the dataset. In our study, the output layer produces five neurons: dyed-lifted-polyps, normal-cecum, normal-pylorus, polyps, and ulcerative-colitis. Neurons are interconnected through weights, and information is transmitted from one neuron to another and from one layer to another in each repetition [38]. The algorithm is characterized by many interconnected neurons, the interconnected processing unit, and the activation and bias function associated with each neuron. It is characterized by the basis of learning and calculating the error rate in each iteration. The error rate is calculated between each iteration’s actual and predicted output. The process continues, and in each iteration, the weights are updated until the minimum sum squared (MSE) is obtained between the actual and predicted output, as described by Equation (20).

(20) $$\;\mathrm{MSC}=\frac{1}{n}\overset{n}{\underset{i=1}{\sum }}\;{\left(\;a{\left(x\right)}_{i}-p{\left(y\right)}_{i}\right)}^{2}\;$$

where $a{\left(x\right)}_{i}\;$represents the actual values and $p{\left(y\right)}_{i}$ represents the predicted values.

**Figure 5:**
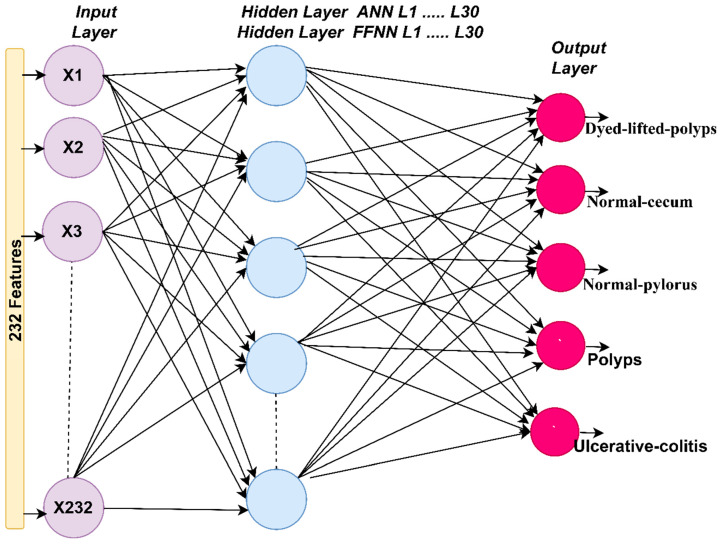
Structure of neural network algorithms for classifying the endoscopy image dataset of lower gastrointestinal diseases.

FFNN is a type of intelligent neural network that consists of three layers, with each layer having many interconnected neurons that forward data. It is distinguished by its superior ability to solve, interpret and analyze many complex problems efficiently [39]. The algorithm consists of three layers: the input, hidden, and output layers. The input layer consists of 232 neurons that receive inputs as features extracted from the feature extraction stage. The algorithm also consists of 30 hidden layers that solve complex problems, analyze, interpret, and send them to the output layer. The output layer consists of five neurons to show the results of classifying each image into its appropriate class as shown in Figure 5. In our study, the algorithm produces five neurons: dyed-lifted-polyps, normal-cecum, normal-pylorus, polyps, and ulcerative-colitis. In this network, information flows between neurons in the forward direction; neurons are interconnected by weights w, and weights are transmitted from one cell to another in the forward direction. Each neuron produces a weight multiplied by the value of the weights of the previous neurons, and the process continues, and with each iteration, the weights are updated. The weights are spoken in each iteration until the minimum sum squared is obtained between the actual and expected output described by the above equation.

### 3.4. The Second Proposed System (CNN Models)

Convolutional neural network (CNN) techniques are compatible with machine learning techniques, and what distinguishes them is the use of many deep layers to analyze and interpret the required tasks with high accuracy and efficiency [40]. CNN models are called deep learning due to the use of deep convolutional layers and the essence of their work is to obtain a representation of many levels to represent the problem starting from simple modular levels that transform the required tasks (endoscopic images of lower GI) from levels to more deeper levels to extract features deeper and more abstract. CNNs are used in classification, regression, texture modelling, image recognition, natural language processing, biomedical image classification, robotics, and other tasks. In classification tasks, networks amplify representation layers to extract the essential features to distinguish each image into its appropriate class and suppress irrelevant differences. CNN architecture refers to many non-linear levels that learn something specific in each layer; for example, one layer works to extract geometric features, while another layer focuses on extracting color features, a layer for texture features, another layer for showing edges, and so on. A CNN consists of many layers, the most important of which are the convolutional layers, the pooling layer, the fully connected layer, and many auxiliary layers.

Convolutional layers are one of the most important layers in CNN models and derive their name from convolutional neural networks. Three parameters that control convolutional layers are filter size, zero-padding, and p-step. The filters work on the process of convolution between *w*(*t*) and the target images *x*(*t*), which is called the process of convolution (x × w)(t) or *s*(*t*) as shown in Equation (21). The larger the filter size, the more wrap around the image. Each filter has a specific function, some work to discover edges, some filters to extract geometry features, and some to extract texture, shape, color features, and so on. For zero-padding, which preserves the size of the original image, the edges of the image are padded with zeros, and the size of zero-padding is determined based on the size of the filter convoluting around the original image [41]. For p-step, determine the number of steps the filter can move around the image.

(21) $$s\left(t\right)=\left(x\times w\right)\left(t\right)=\int_{-\infty }^{\infty }x\left(a\right)w\left(t-a\right)\;da$$

where (x × w) (t) is the process of convolution, *x*(*a*) is the image to be processed, and *w*(*t* − *a*) is the filter convolted around the image.

Pooling layers are one of the primary layers that reduce the dimensions of the input image. After convolutional operations, it produces millions of parameters and thus slows down the training process. Therefore, to represent the high-dimensional data space in the low-dimensional space while preserving the most important features and to speed up the training process, Pooling layers work to solve these challenges. Pooling layers work as convolutional layers, where the size of a filter is determined in the layers Pooling and moving on the image and the representation of groups of pixels by one pixel based on the methods Max-Pooling and Average-Pooling. The Max-Pooling mechanism identifies pixels and represents them by the maximum value [42]. Equation (22) describes the mechanism of Max-Pooling. While in the Average-Pooling method, groups of pixels are selected, their average value is calculated, and groups of pixels are represented by their average value. Equation (23) describes how the Average-Pooling method works.

(22) $$P\left(i;\;j\right)=max_{m,n=1\ldots .k}\;A\left[\left(i-1\right)p+m;\;\left(\;j-1\right)p+n\right]$$

(23) $$P\left(i;\;j\right)=\frac{1}{k^{2}}\underset{m,n=1\ldots .k}{\sum }A\left[\left(i-1\right)p+m;\;\left(\;j-1\right)p+n\right]\;$$

where *A* is filter size, *m*,*n* are filtered size dimensions, *p* is filtered step size, *k* is capacity of filter.

Fully Connected Layers (FCL) is one of the primary layers in CNN and is responsible for classifying input images into their appropriate classes. The FCL comprises millions of neurons connected by junctions called weights. FCL converts the dimensions of deep feature maps from 2D to 1D. Some CNN models have many FCL layers. Finally, the FCL layer sends its output to the Softmax activation function, producing neurons with the number of classes in the dataset. In our study, the Softmax layer produces five neurons: dyed-lifted-polyps, normal-cecum, normal-pylorus, polyps, and ulcerative-colitis. Equation (24) describes how the Softmax activation function works.

(24) $$y\left(x_{i}\right)=\frac{\exp x_{i}}{\;\sum_{j=1}^{n}\;\exp x_{j}}\;$$

*y(x)* between 0 ≤ *y(x)* ≤ 1.

There are also many auxiliary layers such as Rectified Linear Unit (ReLU), dropout layer, and others. ReLU follows convolutional layers to process the output of convolutional layers. This layer passes only positive outputs, while converting negative outputs to zeros. Equation (25) describes the working mechanism of the ReLU layer.

(25) $$\mathrm{ReLU}\left(x\right)=\max\left(0,\;x\right)=\left\{\begin{array}{c} x,\;x\geq 0\\ 0,\;x<0 \end{array}\right.\;$$

Convolutional layers produce millions of operands, and thus networks experience overfitting. Therefore, CNNs provide a dropout layer to solve these challenges. The dropout layer passes a set number of neurons on each iteration. In this study, CNNs models pass 50% of the neurons in each iteration, but this layer will double the training time of the dataset.

This study will focus on two models, GoogLeNet and AlexNet.

#### 3.4.1. GoogLeNet Model

**Figure 6:**
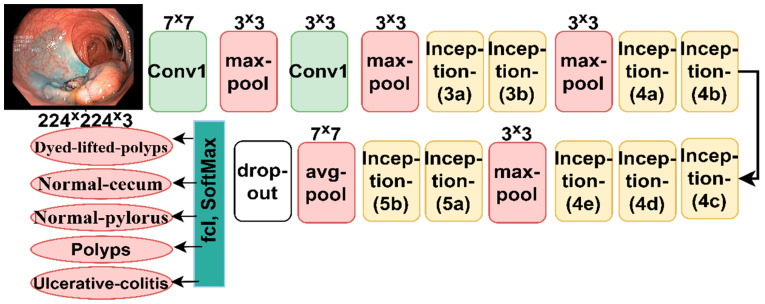
General structure of the GogLeNet model for classifying the lower GI endoscopy image dataset.

GoogLeNet is a convolutional neural network used in many applications for pattern recognition and classification purposes, including biomedical image processing. GoogLeNet consists of 27 layers. This model is distinguished from other models in that it contains layers that can significantly reduce the dimensions while preserving the essential features. The network has a convolutional layer with a 7 × 7 filter that quickly extracts feature maps. The network also contains three 3 × 3 pooling layers, which can reduce the dimensions and reduce the width and height of the image; Additionally, the network has a pooling layer of 7 × 7 size which greatly reduces the dimensions and is effective while preserving the essential features [41]. All layers of the network produce seven million parameters. Figure 6 shows the GoogLeNet architecture for classifying endoscopy images of the lower GI dataset.

#### 3.4.2. AlexNet Model

**Figure 7:**
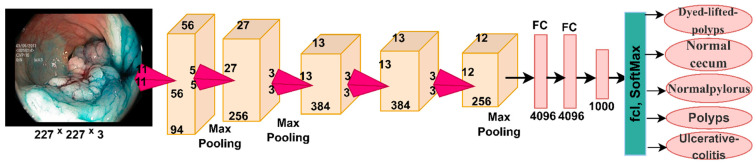
General structure of the AlexNet model for classifying the lower GI endoscopy image dataset.

AlexNet is a type of convolutional neural network that contains many deep layers. AlexNet consists of 25 layers divided between convolutional, pooling, fully connected, and helper layers. The first layer is the input layer, which receives the endoscopy images of the lower GI dataset and adjusts the size of all images to a uniform size of 227 × 227 × 3. There are five convolutional layers for feature map extraction, ReLU layers for further feature processing, and three pooling layers from the max-pooling layers that reduce dimensions; two dropout layers to overcome the problem of overfitting; and three fully connected layers with softmax activation function [43]. AlexNet produces 62 million parameters and 650,000 neurons interconnected by 630 million connections. Figure 7 shows the structure of the AlexNet model to classify the lower GI dataset.

### 3.5. The Third Proposed System (Hybrid of Deep Learning and Machine Learning)

**Figure 8:**
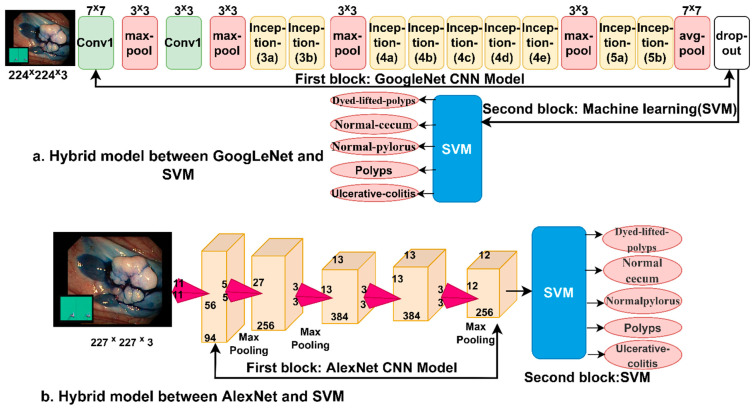
The general structure of the hybrid method between CNN models and SVM (**a**) GoogleNet+SVM and (**b**) AlxNet+SVM.

In this section, a new technique is introduced, a hybrid between CNN modelling techniques and a machine learning algorithm for classifying endoscopic images of the lower GI dataset. Since CNN models require high-resource and expensive computer specifications and take a long time to train the dataset, these hybrid techniques solve these challenges [44]. Therefore, in this study, a hybrid technique is presented between (GoogLeNet and AlexNet) models and the SVM machine learning algorithm, which requires medium computer resources and is fast to train the data and produces high performance. The basic idea in this technique consists of two blocks; the first block is CNN models that extract the maps of the deep features of the endoscopic images and send them to the second block. The second block is an SVM algorithm that receives deep feature maps and classifies them with high accuracy and efficiency. Figure 8a,b shows the basic structure of the hybrid technique, where it is noted that the technique contains two blocks are CNN models (GoogLeNet and AlexNet). The second block is the SVM algorithm, and therefore the technique is called GoogLeNet+SVM and AlexNet+SVM. It is worth noting that the fully connected layer was removed from the CNN models and replaced with the SVM algorithm.

### 3.6. The Fourth Proposed System (Hybrid Features)

**Figure 9:**
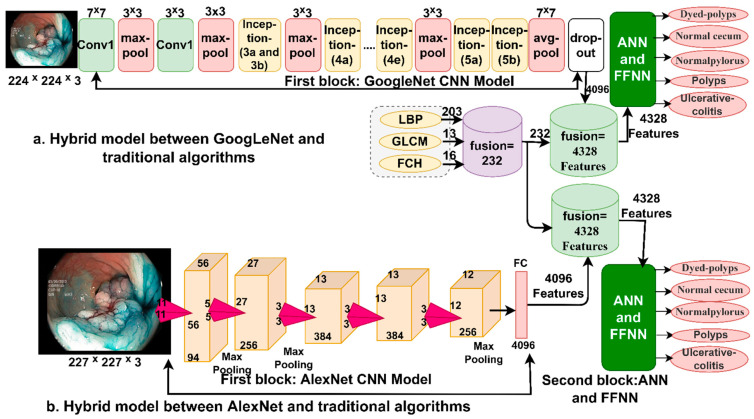
The general structure of feature fusion between CNN models and LBP, GLCM, and FCH algorithms.

This section presents modern methods using hybrid techniques between deep feature maps extracted by CNN models and features extracted by traditional algorithms. CNN models require high specification and expensive computer resources and take a long time to train the dataset. Therefore, the hybrid features are classified using the ANN and FFNN algorithms to solve these challenges [45]. The main idea of this technique is as follows: First, extract the deep feature maps from GoogLeNet and AlexNet models and store them in feature vectors, where 4096 features are extracted for each image from each model. Second, combine the features extracted from the LBP, GLCM and FCH algorithms, producing 232 features after fusing them. Third, the deep feature maps were converted into a unified data format. Fourth, the deep feature maps extracted from CNN models (the third step) are combined with the features extracted by traditional algorithms (the second step), so after fusion, it produced 4328 features representing each image (vector). Fifth, these features are fed to the ANN and FFNN algorithms to classify them. Figure 9 shows the methodology for extracting deep feature maps by GoogLeNet and AlexNet and combining them with the features extracted by LBP, GLCM and FCH and then fed to ANN and FFNN algorithms.

## 4. Experimental Results

### 4.1. Splitting Dataset

In this study, endoscopic images of the lower gastrointestinal disease dataset were evaluated by four proposed systems, each with more than one algorithm. There are various methods of dataset analysis using neural network algorithms, CNN models, SVM, and hybrid methods for classifying and extracting hybrid features. The dataset contains 5000 images equally divided into five types of diseases: dyed-lifted-polyps, normal-cecum, normal-pylorus, polyps, and ulcerative-colitis. The dataset was divided into 80% for training and validation (80:20) and 20% for testing, as described in Table 1. All the proposed systems in this study were implemented using MATLAB 2018b environment and with Intel^®^ i5 processor specifications, RAM 12GB and GPU 4GB.

### 4.2. Evaluation Metrics

In this study, the lower GI dataset was evaluated by several systems proposed, which are neural networks (ANN and FFNN), CNN models (GoogLeNet and AlexNet), hybrid method between CNN models and SVM (GoogLeNet+SVM, AlexNet+SVM), and hybrid features extracted between CNN models (GoogLeNet and AlexNet) and algorithms (LBP, GLCM and FCH) by many statistical measures. The proposed systems were evaluated by using the same scales which are accuracy, precision, sensitivity, specificity, and AUC described in Equations (26)–(30). All the proposed systems produced a confusion matrix that contains all the test samples that are classified as correct and incorrect. Correctly labeled samples are called true positive (TP) for confirmed samples and true negative (TN) for healthy samples. Incorrectly labeled samples are called false negative (FN) and false positive (FP) [46].

(26) $$\mathrm{Accuracy}=\frac{\mathrm{TN}+\mathrm{TP}}{\mathrm{TN}+\mathrm{TP}+\mathrm{FN}+\mathrm{FP}}\times 100\%$$

(27) $$\mathrm{Precision}=\frac{\mathrm{TP}}{\mathrm{TP}+\mathrm{FP}}\times 100\%\;$$

(28) $$\mathrm{Sensitivity}=\frac{\mathrm{TP}}{\mathrm{TP}+\mathrm{FN}}\times 100\%\;$$

(29) $$\mathrm{Specificity}=\frac{\mathrm{TN}}{\mathrm{TN}+\mathrm{FP}}\times 100\;$$

(30) $$\mathrm{AUC}\;=\frac{\mathrm{True}\;\mathrm{Positive}\;\mathrm{Rate}}{\mathrm{False}\;\mathrm{Positive}\;\mathrm{Rate}}=\frac{\mathrm{Sensitivity}}{\mathrm{Specificity}}$$

where TP is the number of properly classified GI endoscopy images of as diseases. TN is the number of GI endoscopy images correctly classified as normal. FP is the number of endoscopy images of a normal GI tract but it is classified as diseases. FN is the number of endoscopic images of GI diseases classified as normal.

### 4.3. Segmentation Performance Evaluation

The segmentation method is one of the most important steps to biomedical image processing, which is widely used in this field to select the affected region and separate it from the rest of the image. In this study, the Active Contour method was applied, which acts as a snake movement and starts with a point and moves along the edges of the lesion until the region of interest (the lesion region) is completely selected, then separated it from the rest of the image. The segmentation method based on Active Contour models was validated by accuracy, precision and recall measures, which reached 99.3%, 99.7%, and 99.6%, respectively. Thus, the lesion region was separated with a promising accuracy and high efficiency, and the region of interest was sent to the feature extraction stage, to extract the most important color, texture, and shape features.

### 4.4. Results of the First Proposed System (Neural Networks Algorithms)

**Figure 10:**
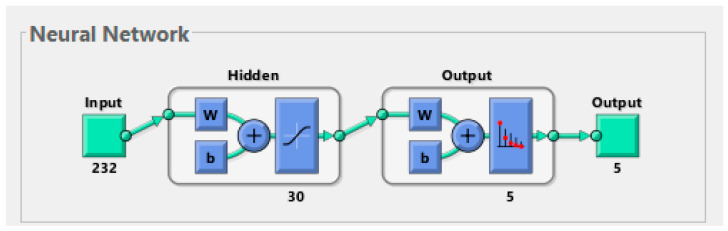
Display performance of the ANN and FFNN algorithms for training a low GI dataset.

In many domains, including medical image diagnosis, neural networks are among the most important and successful artificial intelligence networks. The final stage of categorization is neural network algorithms, which are based on their efficiency in previous stages of image processing (pre-processing, segmentation and feature extraction). Endoscopic images of lower gastrointestinal illnesses were classified in this work using the algorithms ANN and FFNN, which were fed the features derived by the hybrid technique. The dataset was separated into two parts: 80 percent for training and validation and 20% for testing. Figure 10 shows the process of training the ANN and FFNN networks, where it is noted that the network consists of an input layer with 232 neurons, 30 hidden layers for both ANN and FFNN, and an output layer with five neurons; each neuron represents one of the classes of the dataset.

#### 4.4.1. Gradient

**Figure 11:**
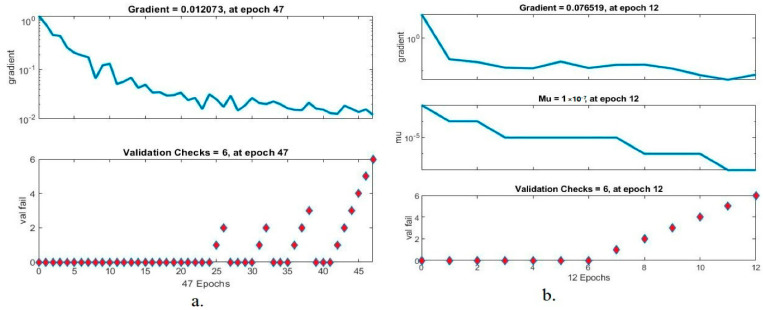
Displays gradient and validation value of lower GI dataset using (**a**) ANN (**b**) FFNN.

There are many methods for assessing the lower GI dataset using the ANN and FFNN algorithms, and one of these scales is the gradient values. The gradient value measures the error rate between actual and predicted values. Figure 11a,b shows the gradient values and validation check for the performance of the ANN and FFNN algorithms for evaluating the lower GI dataset. It can be seen from Figure 11a that the dataset was evaluated by ANN, which found the best gradient at a value of 0.012073 at epoch 47 and validated at a value of 6 during the same epoch. It can also be seen from Figure 11b that the dataset was evaluated by the FFNN algorithm, which reached the best gradation at a value of 0.076519 at epoch 12 and was validated at a value of 6 during the same epoch.

#### 4.4.2. Performance Analysis

**Figure 12:**
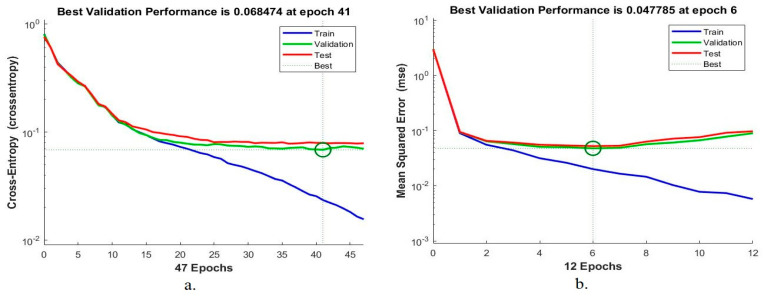
Displays performance for evaluating a lower GI dataset using (**a**) ANN (**b**) FFNN.

Cross-entropy loss is one of the performance measures of ANN and FFNN, which measures mean squared error between actual and predicted values. Figure 12 shows the performance of the ANN and FFNN networks for assessing the lower GI dataset during the training, validation and testing phases. It is noticed from the figure that the crossed lines represent the best point reached by the algorithms, and the blue line is for the training stage, green for the validation stage and red for the testing stage. The ANN algorithm achieved the best performance with a value of 0.068474 at epoch 41, as shown in Figure 12a. The FFNN algorithm achieved the best performance with a value of 0.047785 at epoch 6, as shown in Figure 12b. When the validation stage reaches optimal performance, the network parameters are adjusted, and the network stops training.

#### 4.4.3. Receiver Operating Characteristic (ROC)

**Figure 13:**
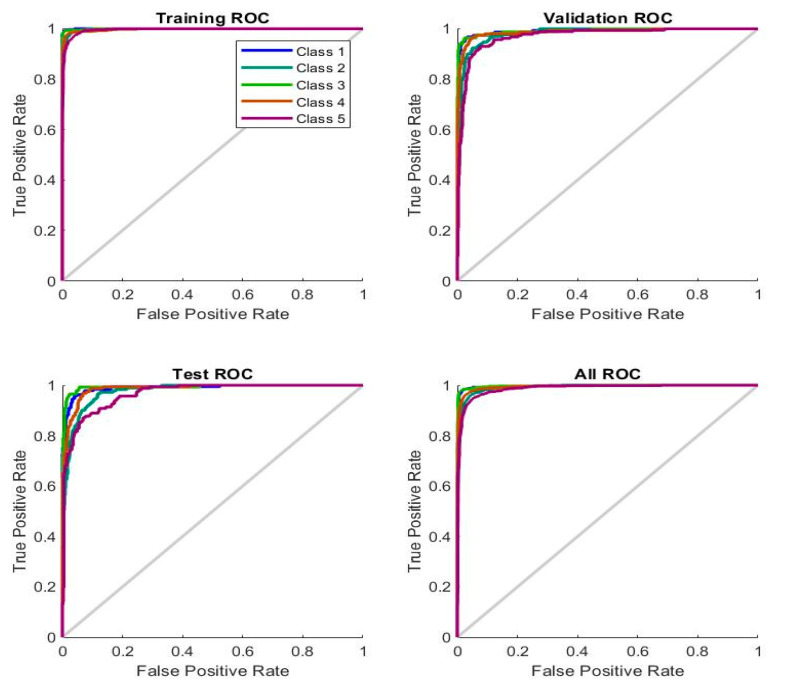
Displays the performance of ANN through ROC for endoscopic image diagnosis.

ROC is one of the performance measures of neural networks to evaluate their performance on the lower GI dataset. ROC is measured by measuring the positive and false samples rate ratio during the training, validation, and testing phases. Figure 13 describes the performance of the ANN network for assessing the lower GI dataset, where the *x*-axis represents samples for the false positive rate (FPR) with specificity. In contrast, the *y*-axis represents samples for the true positive rate (TPR) labelled with sensitivity. It is noted from the figure that there are five colors; each color represents a class in the dataset. ANN achieved an overall ROC of 98.82% for all five types of diseases in the dataset.

#### 4.4.4. Regression

**Figure 14:**
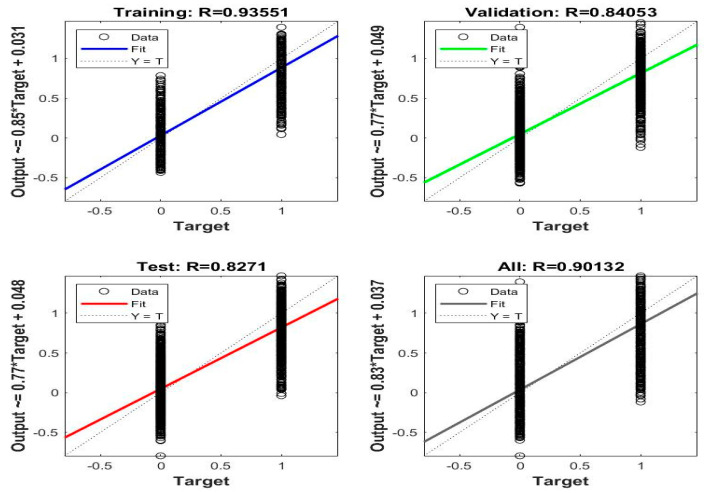
Displays the performance of FFNN through regression values for endoscopic image diagnosis.

Regression is one of the performance measures of neural networks for evaluating a dataset. The network finds the regression value by predicting continuous variables through other available variables by measuring the error rate between the target and output values. The network finds the best value for the regression when it approaches the value 1, which means that the error rate between the target and output values is zero. Figure 14 shows the performance of the FFNN for evaluating the regression of the dataset and predicting continuous values according to the available values. FFNN reached a regression of 93.55% during the training phase and 84% during the validation phase, and during the testing phase, it reached 82.71% and achieved an overall regression of 90.13%.

#### 4.4.5. Error Histogram

**Figure 15:**
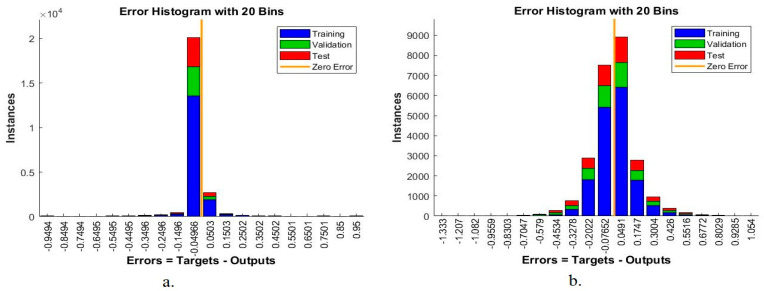
Displays error histogram bin for evaluating a lower GI dataset using (**a**) ANN (**b**) FFNN.

The error histogram measures the performance of the ANN and FFNN algorithms on the lower GI dataset. The ANN and FFNN algorithms find the minimum error between the actual and predicted output during the dataset’s training, validation, and testing phase. Figure 15 describes the performance of the ANN and FFNN algorithms on the dataset, where the blue histogram bin is represented during the training phase, the green histogram bin is during the validation phase, the red histogram bin is during the test phase of the dataset, and the orange line is the zero line between the actual and predicted values. Figure 15a shows the performance of the ANN algorithm, which reached the minimum error between the actual and predicted values at 20 bin between the values −0.9494 and 0.95. While Figure 15b shows the performance of the FFNN algorithm, which reached the minimum error between the actual and predicted values at 20 bin between the values −1.333 and 1.054.

#### 4.4.6. Confusion Matrix

**Figure 16:**
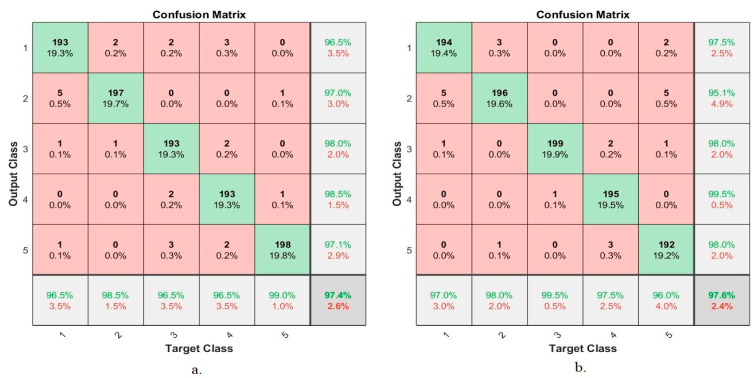
Confusion matrix for the low GI dataset generated by using (**a**) ANN (**b**) FFNN.

The confusion matrix is the comprehensive and most important measure for evaluating networks on a dataset. It is a matrix-like form in which a row and a column represent each class (disease) of the dataset. The rows represent the (actual) output images, while the columns represent the predicted images. The confusion matrix contains all dataset samples that are correctly and incorrectly classified. Correctly classified samples are called true positive (TP) and true negative (TN); incorrectly labelled samples are called false positive (FP) and false negative (FN). In this study, endoscopic images of the lower GI dataset were evaluated by ANN and FFNN during the training, validation and testing phase. Figure 16 shows the resulting confusion matrix from ANN and FFNN algorithms representing the evaluation of the dataset for five diseases as follows: class 1 represents dyed-lifted-polyps, class 2 represents normal-cecum, class 3 represents normal-pylorus, class 4 represents polyps, and class 5 represents ulcerative-colitis. Figure 16a shows the performance of the ANN algorithm, which reached an overall accuracy of 97.4%. Figure 16b also shows the performance of the FFNN algorithm, which reached an overall accuracy of 97.6%.

**Figure 17:**
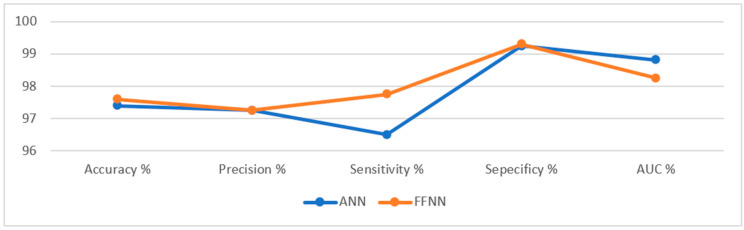
Display of the performance of the ANN and FFNN algorithms for diagnosing a low GI dataset.

Table 2 summarizes the results of the evaluation of the ANN and FFNN algorithms on endoscopic images for the early diagnosis of lower gastrointestinal disease. It is noted that the FFNN algorithm is superior to the ANN algorithm. The ANN algorithm achieved an accuracy of 97.4%, a precision of 97.25%, a sensitivity of 96.5%, a specificity of 99.25%, and an AUC of 98.82%. In contrast, the FFNN algorithm achieved an accuracy of 97.6%, precision of 97.25%, the sensitivity of 97.75%, specificity of 99.3%, and AUC of 98.25%. Figure 17 presents the performance of the ANN and FFNN algorithms for evaluating the lower GI dataset.

### 4.5. Results of Second Proposed System (CNN Models)

In this section, the endoscopic image of the lower GI dataset is evaluated using the pre-trained CNN models, GoogLeNet and AlexNet. Transfer learning method is pre-trained CNN models on more than one million images to produce more than a thousand classes. Thus, the performance of the experience of the previously trained models is transferred to perform new tasks, as in this study, where the experience of the CNN models for diagnosing lower GI dataset is transferred. One of the challenges facing CNN models is the overfitting problem during the training phase of the dataset. Thus, CNN models introduce the data augmentation technique to overcome this challenge, which artificially augments dataset images. Table 3 summarizes the lower GI dataset before and after using the data augmentation during the training phase. Images are artificially augmented through many operations such as rotation, flipping, shifting, and others. Each image was incremented seven times for all classes equally.

Table 4 summarizes the tuning of the CNN GoogLeNet and AlexNet models, where the adam optimizer and Mini Batch Size, Mini Batch Size, Initial Learn Rate, dataset training time for each model and Validation Frequency were set.

The GoogLeNet and AlexNet models achieved superior results for diagnosing endoscopic images of the gastro-intestinal disease dataset. Table 5 describes the evaluation results of the GoogLeNet and AlexNet models, where it is noted that the GoogLeNet model is superior to the AlexNet model. The GoogLeNet model achieved an accuracy of 96%, a precision of 96.2%, a sensitivity of 96%, a specificity of 99.2%, and an AUC of 96%. In contrast, the AlexNet model achieved an accuracy of 91.5%, a precision of 91.8%, a sensitivity of 91.4%, a specificity of 98%, and an AUC of 99.53%.

**Figure 18:**
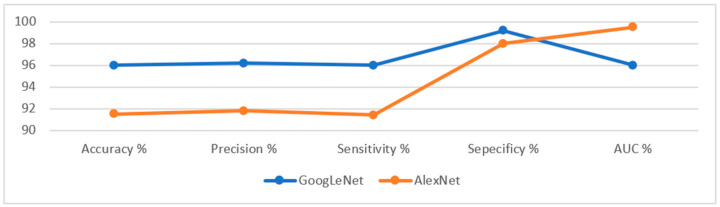
Display results of the GoogLeNet and AlexNet models for diagnosing a low GI dataset.

Figure 18 presents the evaluation results of the performance of the GoogLeNet and AlexNet models on the lower GI dataset in a graph.

**Figure 19:**
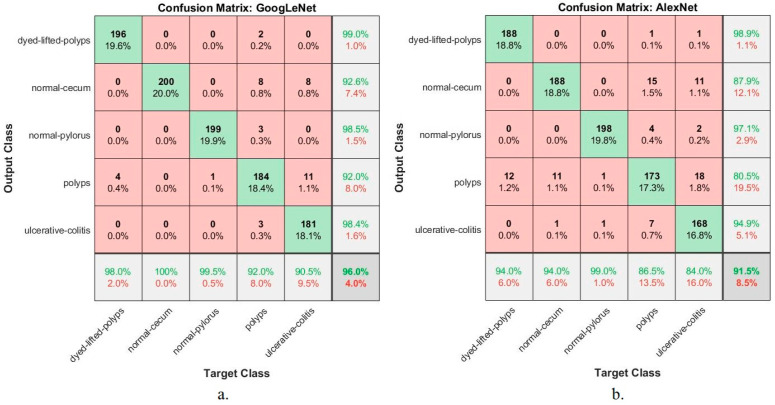
Confusion matrix for the lower GI dataset generated by using (**a**) GoogLeNet (**b**) AlexNet.

Figure 19 describes the confusion matrix generated by the CNN models, GoogLeNet and AlexNet for the early diagnosis of lower GI disease. In contrast, the confusion matrix describes all dataset samples that are correctly or incorrectly categorized. It also describes the diagnostic accuracy reached by the models for each class. The figure shows that dyed-lifted-polyps was diagnosed with 98% and 94% accuracy for GoogLeNet and AlexNet, respectively. Normal-cecum was diagnosed with 100% and 94% accuracy for GoogLeNet and AlexNet, respectively. Normal-pylorus was diagnosed with 99.5% and 99% accuracy for GoogLeNet and AlexNet, respectively. Polyps were diagnosed with an accuracy of 92% and 86.5% for GoogLeNet and AlexNet, respectively. Ulcerative colitis was diagnosed with an accuracy of 90.5% and 84% for GoogLeNet and AlexNet, respectively.

### 4.6. Results of Third Proposed System (Hybrid CNN with SVM)

This section presents the findings of the hybrid techniques between CNN models (GoogLeNet and AlexNet) and the SVM algorithm. The technique consists of two blocks: the first is CNN models for extracting feature maps, and the second block is the SVM algorithm for classifying feature maps. One of the most important reasons for using this technique is that it requires medium-specification computer resources, speed in training the dataset, and high accuracy in diagnosis. Table 6 summarizes the assessment of the lower gastrointestinal diseases dataset by hybrid GoogLeNet+SVM and AlexNet+SVM technique for early diagnosis of gastrointestinal tumors and ulcers. The GoogLeNet+SVM hybrid technique is superior to AlexNet+SVM. The GoogLeNet+SVM achieved an accuracy of 96.7%, a precision of 96.8%, a sensitivity of 96.8%, a specificity of 99%, and an AUC of 99.1%. In contrast, the AlexNet+SVM model achieved an accuracy of 94.7%, a precision of 94.8%, a sensitivity of 94.8%, a specificity of 98.6%, and an AUC of 99.6%.

**Figure 20:**
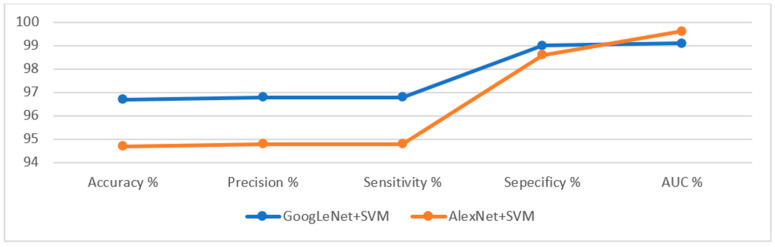
Display results of the GoogLeNet+SVM and AlexNet+SVM techniques for diagnosing a low GI dataset.

Figure 20 displays the evaluation results of the GoogLeNet+SVM and AlexNet+SVM techniques on the lower GI dataset in a graph.

**Figure 21:**
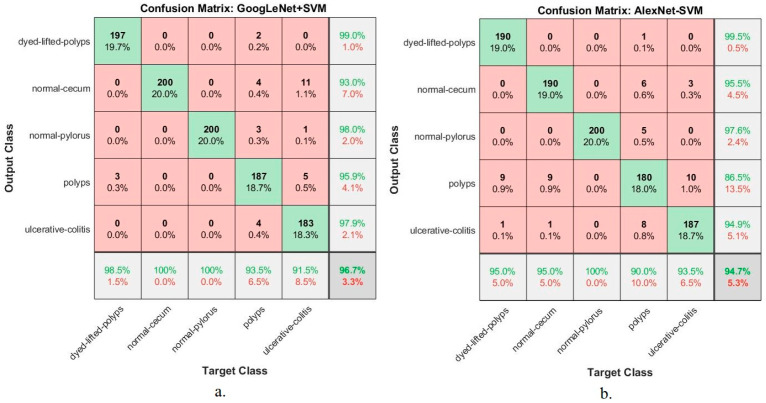
Confusion matrix for evaluating of lower GI dataset using (**a**) GoogLeNet+SVM and (**b**) AlexNet+SVM.

Figure 21 shows the performance of the hybrid techniques GoogLeNet+SVM and AlexNet+SVM for diagnosing lower gastrointestinal disease dataset in the form of a confusion matrix. The hybrid methods produced a confusion matrix that describes all samples of the dataset correctly labelled represented in the primary diameter and all samples incorrectly classified and distributed over the rest of the matrix cells. The figure shows the performance of hybrid techniques for diagnosing each disease and the overall accuracy. The figure shows that dyed-lifted-polyps was diagnosed with 98.5% and 95% accuracy for GoogLeNet+SVM and AlexNet+SVM, respectively. Normal-cecum was diagnosed with 100% and 95% accuracy for GoogLeNet+SVM and AlexNet+SVM, respectively. Normal-pylorus was diagnosed with 100% and 100% accuracy for GoogLeNet+SVM and AlexNet+SVM, respectively. Polyps were diagnosed with an accuracy of 93.5% and 90% for GoogLeNet+SVM and AlexNet+SVM, respectively. Ulcerative colitis was diagnosed with an accuracy of 91.5% and 93.5% for GoogLeNet+SVM and AlexNet+SVM, respectively.

### 4.7. Results of Fourth Proposed System (Hybrid Features CNN and Traditional Algorithms)

This section presents the evaluation results of hybrid feature techniques between CNN models (GoogLeNet and AlexNet) and features extracted by traditional algorithms (LBP, GLCM and FCH); after fusion, all the features are classified by ANN and FFNN algorithms. These techniques require low-resource computer specifications, execution speed, and high accuracy in diagnosing endoscopic images of the lower GI dataset. Table 7 summarizes the evaluation results of the performance of the ANN algorithm. When using the hybrid features extracted by CNN models and traditional algorithms (LBP, GLCM and FCH), the systems reached superior results in diagnosing the lower GI dataset. All features are fused into a single feature vector for each image, where each feature vector contains 4328 features fed into the ANN and FFNN classifiers.

First, when diagnosing by ANN algorithm based on the combined features of GoogLeNet and traditional algorithms (LBP, GLCM and FCH), the system reached accuracy, precision, sensitivity, specificity and AUC of 98%, 98.25%, 97.8%, 99.4% and 98.69%, respectively. When using the hybrid features between AlexNet and traditional algorithms (LBP, GLCM and FCH), the system reached accuracy, precision, sensitivity, specificity and AUC with a percentage of 99.1%, 99%, 98.8%, 99.8% and 99.76%, respectively.

Second, when diagnosing by FFNN algorithm based on the combined features of GoogLeNet and traditional algorithms (LBP, GLCM and FCH), the system reached accuracy, precision, sensitivity, specificity and AUC of 98.8%, 98.6%, 98.2%, 99.75% and 98.83%, respectively. When using the hybrid features between AlexNet and traditional algorithms (LBP, GLCM and FCH), the system reached accuracy, precision, sensitivity, specificity and AUC with a percentage of 99.3%, 99.2%, 99%, 100% and 99.87%, respectively.

**Figure 22:**
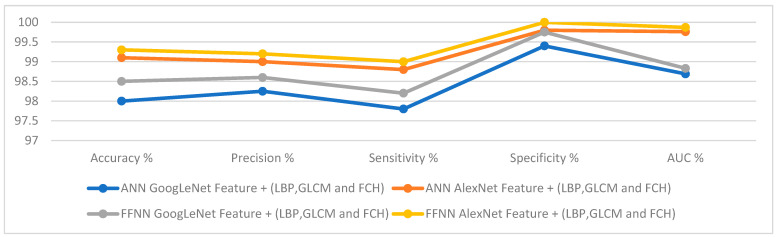
Display of the performance of the ANN and FFNN based on hybrid features for diagnosing a low GI dataset.

Figure 22 displays the evaluation of the ANN and FFNN algorithms based on the fusion of features between CNN models and traditional algorithms to classify the lower GI dataset accurately.

**Figure 23:**
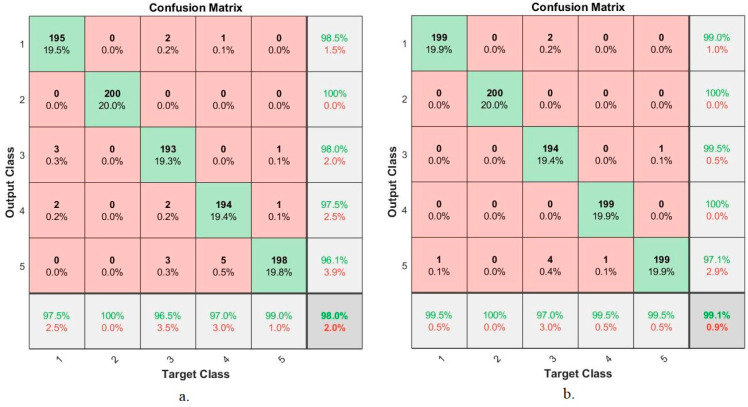
Performance results of the ANN for diagnosing lower gastrointestinal diseases based on hybrid features (**a**) GoogLeNet with traditional algorithm and (**b**) AlexNet with traditional algorithm.

Figure 23 shows the evaluation results of the ANN algorithm based on the hybrid features between CNN models (GoogLeNet and AlexNet) with the features extracted by LBP, GLCM and FCH methods for early diagnosis of lower gastrointestinal diseases. The figure summarizes all samples of the correctly classified and incorrectly classified dataset and displays the diagnostic accuracy of each class (disease) in the dataset. First, when using hybrid features extracted from GoogLeNet and conventional, ANN reached an accuracy of 95.4%, 98.5%, 99.5%, 99.5%, and 100% for diagnosing dyed-lifted-polyps, normal-cecum, normal-pylorus, polyps, and ulcerative-colitis, respectively. Second, when using the hybrid features extracted from AlexNet and conventional, ANN reached an accuracy of 97.2%, 99%, 100%, 99.4%, and 100% for diagnosing dyed-lifted-polyps, normal-cecum, normal-pylorus, polyps, and ulcerative-colitis, respectively.

**Figure 24:**
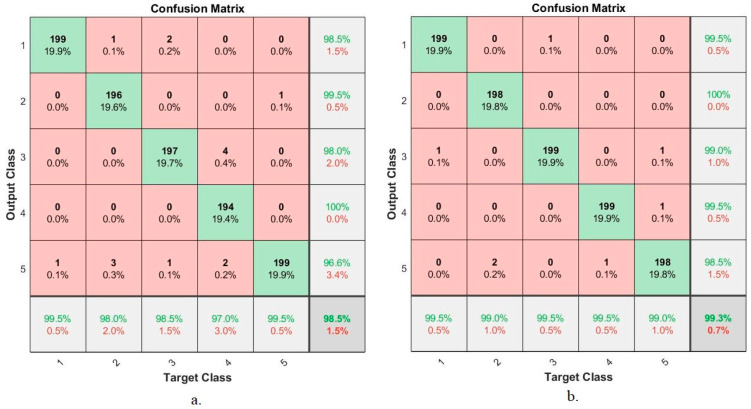
Results of the FFNN for diagnosing lower gastrointestinal diseases based on hybrid features (**a**) GoogLeNet with traditional algorithm and (**b**) AlexNet with traditional algorithm.

Figure 24 shows the confusion matrix produced by the FFNN algorithm based on the hybrid features between CNN models (GoogLeNet and AlexNet) with the features extracted by LBP, GLCM and FCH methods for early diagnosis of lower gastrointestinal diseases. The figure summarizes all samples of the correctly classified and incorrectly classified dataset and displays the diagnostic accuracy of each class (disease) in the dataset. First, when using hybrid features extracted from GoogLeNet and conventional, FFNN reached an accuracy of 99.5%, 98%, 98.5%, 97%, and 99.5% for diagnosing dyed-lifted-polyps, normal-cecum, normal-pylorus, polyps, and ulcerative-colitis, respectively. Second, when using the hybrid features extracted from AlexNet and conventional, FFNN reached an accuracy of 99.5%, 99%, 99.5%, 99.5%, and 99% for diagnosing dyed-lifted-polyps, normal-cecum, normal-pylorus, polyps, and ulcerative-colitis, respectively.

## 5. Discussion and Comparative Analysis

This study presented many methods of artificial intelligence techniques that vary between neural network algorithms, CNN models, hybrid techniques between CNN models, SVM algorithm, and feature merging techniques for early detection of lower GI diseases, which includes 5000 images. Moreover, proposed systems detect and diagnose lower gastrointestinal diseases with a high performance, thus helping to treat and reduce treatment that benefits patients. Clinicians must apply artificial intelligence techniques to diagnose patients and support their diagnostic decisions. The process of data collection and image acquisition from several devices and under different conditions; the influence of external factors such as light reflection; some noise; and internal factors such as mucous membranes and some traces of stool have a negative impact on the diagnostic process, so the average is applied to the three RGB channels. In addition, average and Laplacian filters were applied to enhance the images. Due to the scarcity of medical images, CNN models augment training images by applying the method of data augmentation through flipping, zooming, zooming, and rotating.

Table 8 describes the performance of all proposed systems for diagnosing endoscopic images of the lower gastrointestinal disease dataset. First, for dyed lifted polyps, the ANN algorithm based on fusion features (AlexNet and traditional algorithms) and the FFNN algorithm based on fusion features achieved the best performance for diagnosing this disease with an accuracy of 99.5%.

Second, for Normal-cecum, GoogLeNet, the hybrid technique between GoogLeNet with SVM and the ANN algorithm based on fusion features (GoogLeNet with traditional algorithms and AlexNet with traditional algorithms) achieved the best performance for diagnosing this disease with 100% accuracy. Third, for normal pylorus, the GoogLeNet+SVM and AlexNet+SVM achieved the best performance for diagnosing this disease with 100% accuracy. Fourth, for polyps, the ANN algorithm based on fusion features (AlexNet with traditional algorithms) and the FFNN algorithm based on fusion features (AlexNet with traditional algorithms) achieved the best performance for diagnosing this disease with an accuracy of 99.5%. Fifthly, for ulcerative colitis, the ANN algorithm based on fusion features (AlexNet with traditional algorithms) and the FFNN algorithm based on fusion features (GoogLeNet with traditional algorithms) achieved the best performance for diagnosing this disease with an accuracy of 99.5%.

**Figure 25:**
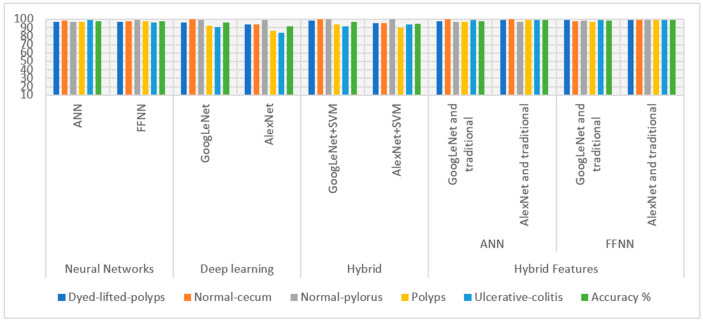
Display of results comparison of all the proposed methods for diagnosing a low GI dataset.

Figure 25 presents the performance of all proposed systems for diagnosing endoscopic images for early detection of lower gastrointestinal diseases in graphic form.

## 6. Conclusions

The increase in deaths due to lower GI diseases, especially tumors, results from the lack of manual diagnosis due to the difficulty in tracking all the frames. Therefore, this study presents a set of proposed multi-method systems for the early diagnosis of endoscopic images of a lower GI dataset. The first proposed system uses the neural networks ANN and FFNN, which are based on segmentation of the region of interest and feature extraction by LBP, GLCM and FCH algorithms and merging them into one feature vector for each image. The second proposed system uses the CNN models GoogLeNet and AlexNet, which are based on extracting deep feature maps and classifying them accurately. The third proposed system uses hybrid techniques based on CNN models (GoogLeNet and AlexNet) to extract deep feature maps and classify them by the SVM algorithm. The fourth proposed system using ANN and FFNN neural network algorithms is based on fused features extracted by CNN models (GoogLeNet and AlexNet) and LBP, GLCM and FCH algorithms. All the proposed systems achieved highly accurate diagnostic results in diagnosing endoscopic images of the lower gastrointestinal disease dataset with high efficiency.

Future work will apply the principal component analysis (PCA) algorithm to reduce the dimensions of deep feature maps extracted by CNN models, in addition to integrating deep feature maps from more than one CNN model and reducing their dimensions by the PCA algorithm.

## Figures and Tables

**Table 1:** Splitting of the endoscopy image of lower GI dataset for training and testing.

Phase	80% for Training and Validation (80:20%)		Testing (20%)
Classes	Training (80%)	Validation (20%)	
dyed-lifted-polyps	640	160	200
normal-cecum	640	160	200
normal-pylorus	640	160	200
polyps	640	160	200
ulcerative-colitis	640	160	200

**Table 2:** The results of the ANN and FFNN algorithms on the gastroenterology dataset.

Measure	ANN	FFNN
Accuracy %	97.4	97.6
Precision %	97.25	97.25
Sensitivity %	96.5	97.75
Specificity %	99.25	99.3
AUC %	98.82	98.25

**Table 3:** Data augmentation method during the training phase.

Phase	Training Phase 80%				
Class name	dyed-lifted-polyps	normal-cecum	normal-pylorus	polyps	ulcerative-colitis
No images before augmentation	640	640	640	640	640
No images after augmentation	**5120**	**5120**	**5120**	**5120**	**5120**

**Table 4:** Seting parameter options for GoogLeNet and AlexNet models.

Options	GoogleNet	AlexNet
training Options	adam	adam
Mini Batch Size	18	120
Max Epochs	6	10
Initial Learn Rate	0.0003	0.0001
Validation Frequency	3	50
Training time (min)	301 min 23 s	144 min 38 s
Execution Environment	GPU	GPU

**Table 5:** The results of the GoogLeNet and AlexNet models on the lower GI dataset.

Measure	GoogLeNet	AlexNet
Accuracy %	96	91.5
Precision %	96.2	91.8
Sensitivity %	96	91.4
Specificity %	99.2	98
AUC %	96	99.53

**Table 6:** The results of the hybrid method on the lower GI dataset.

Measure	GoogLeNet+SVM	AlexNet+SVM
Accuracy %	96.7	94.7
Precision %	96.8	94.8
Sensitivity %	96.8	94.8
Specificity %	99	98.6
AUC %	99.1	99.62

**Table 7:** Results of dataset evaluation by ANN and FFNN based on hybrid features.

Classifiers	ANN		FFNN	
Hybrid Features	GoogLeNet Feature + LBP, GLCM and FCH	AlexNet Feature + LBP, GLCM and FCH	GoogLeNet Feature + LBP, GLCM and FCH	AlexNet Feature + LBP, GLCM and FCH
Accuracy %	98	99.1	98.5	99.3
Precision %	98.25	99	98.6	99.2
Sensitivity %	97.8	98.8	98.2	99
Specificity %	99.4	99.8	99.75	100
AUC %	98.69	99.76	98.83	99.87

**Table 8:** The accuracy achieved by all the proposed systems for evaluating the gastroenterology dataset.

Diseases			Dyed-Lifted-Polyps	Normal-Cecum	Normal-Pylorus	Polyps	Ulcerative-Colitis	Accuracy %
Neural Networks		ANN	96.5	98.5	96.5	96.5	99	97.4
		FFNN	97	98	99.5	97.5	96	97.6
Deep learning		GoogLeNet	96	100	99.5	92	90.5	96
		AlexNet	94	94	99	86.5	84	91.5
Hybrid		GoogLeNet+SVM	98.5	100	100	93.5	91.5	96.7
		AlexNet+SVM	95	95	100	90	93.5	94.7
Hybrid Features	ANN	GoogLeNet and traditional	97.5	100	96.5	97	99	98
		AlexNet and traditional	99.5	100	97	99.5	99.5	99.1
	FFNN	GoogLeNet and traditional	99.5	98	98.5	97	99.5	98.5
		AlexNet and traditional	99.5	99	99.5	99.5	99	99.3

## Data Availability

In this study, the data supporting all the proposed systems were collected by the Kvasir dataset available at this link: https://datasets.simula.no/kvasir/#download (accessed on 30 January 2022).
